# A systematic review of source attribution of human campylobacteriosis using multilocus sequence typing

**DOI:** 10.2807/1560-7917.ES.2019.24.43.1800696

**Published:** 2019-10-24

**Authors:** Alison J Cody, Martin CJ Maiden, Norval JC Strachan, Noel D McCarthy

**Affiliations:** 1 Department of Zoology, University of Oxford, Oxford, United Kingdom; 2 NIHR Health Protection Research Unit in Gastrointestinal Infections, University of Oxford, Oxford, United Kingdom; 3 School of Biological Sciences, University of Aberdeen, St. Machar Drive, Aberdeen, United Kingdom; 4 Warwick Medical School, University of Warwick, Coventry, United Kingdom

**Keywords:** Campylobacter, MLST, source attribution, food-borne infections, bacterial infections, surveillance, epidemiology, molecular methods

## Abstract

**Background:**

*Campylobacter* is a leading global cause of bacterial gastroenteritis, motivating research to identify sources of human infection. Population genetic studies have been increasingly applied to this end, mainly using multilocus sequence typing (MLST) data.

**Objectives:**

This review aimed to summarise approaches and findings of these studies and identify best practice lessons for this form of genomic epidemiology.

**Methods:**

We systematically reviewed publications using MLST data to attribute human disease isolates to source. Publications were from January 2001, when this type of approach began. Searched databases included Scopus, Web of Science and PubMed. Information on samples and isolate datasets used, as well as MLST schemes and attribution algorithms employed, was obtained. Main findings were extracted, as well as any results’ validation with subsequent correction for identified biases. Meta-analysis is not reported given high levels of heterogeneity.

**Results:**

Of 2,109 studies retrieved worldwide, 25 were included, and poultry, specifically chickens, were identified as principal source of human infection. Ruminants (cattle or sheep) were consistently implicated in a substantial proportion of cases. Data sampling and analytical approaches varied, with five different attribution algorithms used. Validation such as self-attribution of isolates from known sources was reported in five publications. No publication reported adjustment for biases identified by validation.

**Conclusions:**

Common gaps in validation and adjustment highlight opportunities to generate improved estimates in future genomic attribution studies. The consistency of chicken as the main source of human infection, across high income countries, and despite methodological variations, highlights the public health importance of this source.

## Introduction

*Campylobacter* gastroenteritis is a leading cause of acute bacterial gastroenteritis in high, low, and middle income countries. The number of confirmed cases has continued to increase across countries of the European Union (214,000 in 2013 to 246,000 in 2016 and 2017) [1], and over 800,000 cases are estimated to occur annually in the United States (data from 2000 to 2008) [2]. In low income countries *Campylobacter* is increasingly implicated in growth faltering among children under 2 years of age [3].

Chicken products have been identified as an important risk factor for human infection by a variety of techniques including natural experiments, case–control studies, and increasingly by the application of genotypic methods [4-10]. Other infection sources identified by observational epidemiological studies include cattle, sheep, pigs, wild birds and the environment [10]. 

Alongside epidemiological studies there has been an increasing use of population genetic analyses to attribute human cases to likely sources. In these analyses, the genetic diversity of isolates from humans is compared with that of collections of *Campylobacter* isolates obtained from possible sources of infection, allowing quantitative attribution to these sources. 

Multilocus sequence type (MLST) data [8] have become the standard data used in such population genetic analyses, the results of which are generally consistent with the findings from epidemiological analyses [11,12]. Large collections of isolates have been sequenced at the MLST loci from a wide range of sources. The approaches provide a potential means of monitoring change in sources of human infection, for example those that occur as a consequence of public health and food chain interventions [13]. Insights obtained from seven-gene MLST analyses can also inform analyses using more extensive genomic data, as large well sampled datasets of whole genome sequenced (WGS) isolates accumulate from humans and putative sources. Other techniques such as multiplex PCR, PFGE, and comparative genomic fingerprinting have neither been taken up widely nor offer compatibility with whole genome based approaches. 

Studies analysing MLST data vary in terms of both the analytical algorithm applied and the reference datasets used [13-18] (‘reference’ data throughout this paper describe data from known reservoirs such as animal species that can act as sources of human infection). Here, our objectives on the use of MLST analysis to attribute infection in human populations to sources are to: (i) summarise the findings from these studies to date; (ii) describe the approaches used; and (iii) identify lessons to guide further genetic source attribution work using these data and more extensive genomic data as they become available.

## Methods

### Search strategy

The literature search strategy aimed to identify articles attributing *Campylobacter* isolates from human infections to possible sources using MLST-based attribution algorithms. Systematic searches were performed on the Scopus, Web of Science, and PubMed databases using a search string comprised of the following terms: ‘campylobacter$’ AND ‘multilocus OR genotype OR genotyping’ AND ‘source$ OR assignment OR attribution’ AND ‘human OR clinical OR disease’. These were carried out on 23 November 2017 and limited to items published from January 2001 onwards, as the *C. jejuni* MLST scheme was first described in this year [8].

The publication lists arising from the three searches were combined and duplicate records removed. Titles and abstracts of the remaining studies were reviewed to ensure that they described the source attribution of clinical *C. jejuni* and/or *C. coli* isolates to potential source populations using an MLST-based algorithm, in English. Complete texts of the final list were then considered to identify whether or not they satisfied the inclusion criteria. Reference sections of these papers were also searched for further candidate publications.

Texts of the resulting article list were scrutinised to identify: (i) datasets used, including their size, geographical origin, and year of disease and potential source isolates; (ii) sample types from which isolates were obtained, e.g. retail chicken meat, cattle faecal sample; (iii) attribution method(s) employed; (iv) loci in the typing scheme; (v) validation, such as self-attribution of isolates from known sources; (vi) adjustment of attribution to correct for identified biases; (vii) proportion of *Campylobacter* clinical isolates attributed to each source.

When relevant details were not in the text, values were calculated from available data or obtained by contacting the authors where possible. In articles that compared results from more than one dataset, for example comparing clinical samples among different years or rural vs urban disease samples, baseline or mean values are reported in this review.

### Statistical analysis

The proportions of human infection with *C. jejuni* attributed to poultry estimated by the two most commonly used algorithms (Asymmetric Island (AI) [17] and STRUCTURE [19]) were compared by the two-group mean comparison test and variation in this proportion across studies described using the I^2^ index from the metaprop command using StataIC 15 (StataCorpLP, Texas).

## Results

**Figure 1:**
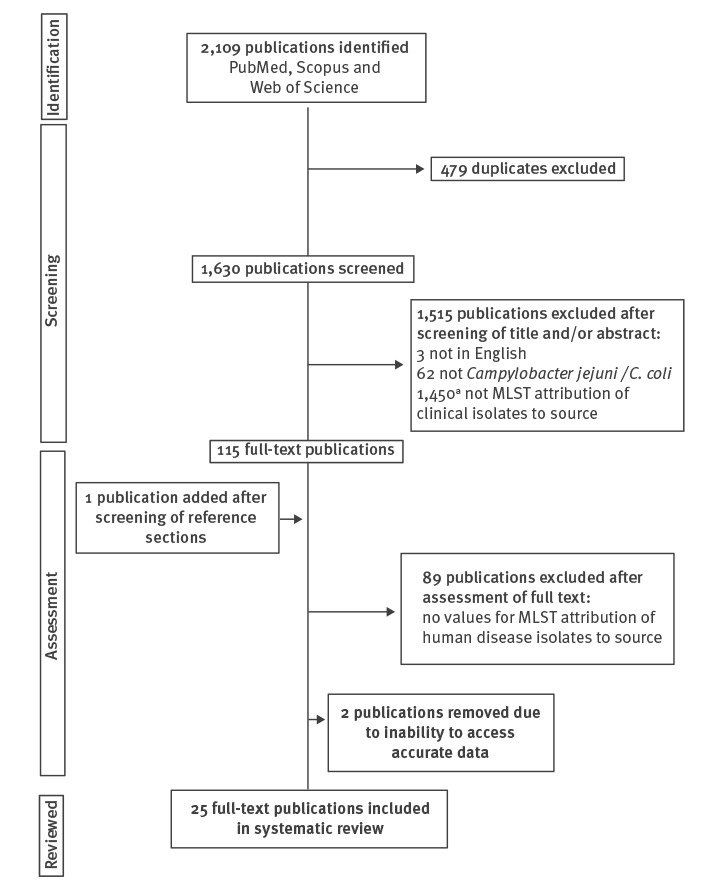
Flow diagram of the search strategy used to identify articles for inclusion in the systematic review****on source attribution of human campylobacteriosis using multilocus sequence typing, since January 2001

Search results and subsequent exclusions, detailed in the preferred reporting items for systematic reviews and meta-analyses (PRISMA) flow diagram (Figure 1) and Table, resulted in 25 articles [13,14,16,17,20-41].

MLST: multilocus sequence typing.

^a ^The search terms were broad and brought in diverse articles with a very diverse corresponding set of reasons for exclusion, including articles that had no relation with source attribution as well as those using other approaches. No useful summary of this diversity was possible.

**Table:** Summary of studies reviewed, indicating composition of reference datasets used for attribution analysis of human *Campylobacter* infections to animal or environmental sources, 2001−2017 (n = 25 studies)

Paper (author/year)	Poultry/chicken	Ruminant/cattle/sheep	Environmental/wild bird dataset(s)	Multiple sample types per source	Number of source populations		Years (max) between clinical and reference isolates
					*C. jejuni*	*C. coli*	
Bessell (2012) [46]	Chicken	Ruminant	Wild bird	Yes	3	NA	16
Boysen (2014) [14]	Poultry	Cattle	NA	Yes	5	NA	1^a^
Cody (2015) [22]	Chicken	Cattle, sheep	Wild bird	No	4	NA	14
Di Giannatale (2016) [23]	Chicken	Cattle, small ruminant	Wild bird, environmental	Yes	6	NA	No data
French (2008) [24]	Chicken	Cattle, sheep	Wild bird, environmental water	Yes	5	NA	3^a^
Jonas (2015) [25]	Chicken	Cattle	NA	Yes	3	3	6
Kittl (2013) [26]	Chicken	NA	NA	Yes	2	2	10^a^
Kovac (2018) [27]	Poultry	Cattle	Environmental (inc. wild birds)	Yes	3	NA	12^a^
Levesque (2013) [28]	Chicken	Cattle	Environmental water, wild bird	No	4	NA	2^a^
Mossong (2016) [29]	Poultry	Ruminant	Environmental water	Yes	4	4	10
Mughini Gras (2012) [30]	Chicken	Cattle, sheep	Environmental (inc. wild birds)	Yes	5	5	13
Mughini Gras (2013) [31]	Chicken	Cattle, sheep	Environmental (inc. wild birds)	Yes	5	5	13
Mullner (2009a) [16]	Chicken	Cattle, sheep	Environmental (inc. wild birds)	Yes	4	NA	3^a^
Mullner (2009b) [13]	Chicken	Cattle, sheep	Environmental (inc. wild birds)	Yes	4	NA	3^a^
Nohra (2016) [32]	Poultry	Ruminant	Environmental water	No	NA	3	5
Rosner (2017) [33]	Chicken	Cattle	NA	Yes	5	5	10
Roux (2013) [34]	Chicken	Cattle, sheep	NA	Yes	NA	4	1^a^
Sears (2011) [35]	Poultry	Cattle, sheep	Environmental water	Yes	4	NA	3^a^
Sheppard (2009) [36]	Chicken	Ruminant	Environmental (inc. wild birds)	Yes	3^b^	5^b^	16
Sheppard (2010) [37]	Chicken	Ruminant	Environmental (inc. wild birds)	Yes	NA	5	6
Smid (2013) [38]	Chicken	Cattle, sheep	Environmental (inc. wild birds)	Yes	4	4	28
Strachan (2009) [39]	Chicken	Cattle, sheep	Wild bird	No	5	5	6
Strachan (2013) [40]	Chicken	Cattle, sheep	Wild bird	No	5	5	2^a^
Thépault (2017) [41]	Chicken	Ruminant	Environmental water	Yes	3	NA	10
Wilson (2008) [17]	Chicken	Cattle and sheep	Wild bird, water, sand	Yes	8	NA	12

Inc.: including; NA: not applicable, whereby this species (*C. jejuni* or *C. coli*) or possible source was not included in the study.

^a^ Clinical and attribution datasets from same temporal range.

^b^ *C. jejuni* results expressed for ruminants; *C. coli* results expressed for cattle and sheep separately.

### Datasets

Twelve papers only investigated *C. jejuni* [13,14,16,17,21-24,27,28,35,41] and three studied only *C. coli* [32,34,37]. Of the 10 publications that considered both *C. jejuni* and *C. coli* [25,26,29-31,33,36,38,39,42], six expressed results by single species [25,26,29,30,33,36] and four reported attribution for the two species jointly [31,38,39,42]. Studies included human clinical isolates from the United Kingdom (n = 9 studies), New Zealand (n = 5), the Netherlands (n = 3), Germany (n = 2), Luxembourg (n = 2), Switzerland (n = 2), Austria, Canada, Denmark, France, Italy and Slovenia (n = 1 study each) (Supplementary Table). 

Fifteen articles reported using reference datasets that combined isolates from more than one potential animal host species or animal species and an environmental reservoir as a single class. Poultry datasets containing predominantly chicken but including other farmed birds were used as an attribution source in five articles [14,27,29,32,35], cattle and sheep isolates combined as ruminants in six reports [21,29,32,36,37,41] and an environmental category comprising at least water and wild bird isolates in 11 [13,16,27,30-32,35-38,43]. A single sample type (e.g. retail meat or faeces) was considered for each host animal species in reference datasets from five studies [22,28,32,39,40] rather than combining isolates across different sample types from the same source.

The highest number of potential source populations to which disease isolates were attributed was eight [17] and the lowest two [26], with reference datasets ranging in size from two [29] to 1,288 [21,36] samples, across the articles reviewed (Supplementary Table). Eleven publications used source isolates from the same time period as human cases [13,16,21,24,26-28,34,35,37,40], with the maximum possible temporal difference between any human and any source isolate ranging between 1 and 12 years in these. The longest time difference between human case and source isolates among the remaining articles was 28 years [38].

One study explicitly considered domestic (with no history of international travel) and travel-associated human cases separately [14] and one compared attribution results of clinical samples from two countries [41]. Reference datasets were limited to the same country as the clinical isolates in 10 studies [13,16,26,28,32,34,35,39,40,43] and an eleventh article compared domestic human case isolates with those of travel-associated cases [14]. Two further publications included reference data from non-domestic sources having established that these countries shared similar disease genotype frequencies as domestic human cases [23,30], and a third compared attribution results using reference MLST data from the same country as the cases and from different countries [38]. The remaining articles used reference isolates from internationally widespread locations.

### Attribution models and data

The studies included used one or more of five attribution algorithms: (i) the Asymmetric Island (AI) model (n = 13 reports) [17]; (ii) STRUCTURE (n = 11) [19]; (iii) the Modified Hald (MH) model (n = 4) [16]; (iv) the Dutch model (n = 2) [44]; and (v) the Hald model (n = 1) [45] (Supplementary Table). Twenty-four of the publications reported data based on the seven housekeeping genes originally described by Dingle [7,8] and a single article identified and used 15 novel host-segregating loci [41]. Clinical datasets composed of both *C. jejuni* and *C. coli,* and those that considered the two species individually, were analysed using either STRUCTURE or AI analysis, whereas studies using the Dutch, Hald, and MH attribution models were restricted to analyses of *C. jejuni* (Supplementary Table). Five of the 25 articles used more than one attribution method [13,14,24,35,36], although one of these only reported results from a single model [35].

### Self-attribution and other validation

Self-attribution estimates the probability that, for example, a chicken-origin isolate will be attributed back to the chicken host reference sample, and repeats this to measure accuracy. These analyses were performed in five of the articles using the AI, STRUCTURE, or both algorithms, and reported average percentage accuracy values for each source tested [26,36-38,41]. For both *C. jejuni* [36], and *C. coli* [26,37,38,41] AI showed greater self-attribution accuracy than the STRUCTURE algorithm. No publication reported adjustment of subsequent attribution of isolates from human cases based on bias identified in self-attribution.

### Attribution results

**Figure 2:**
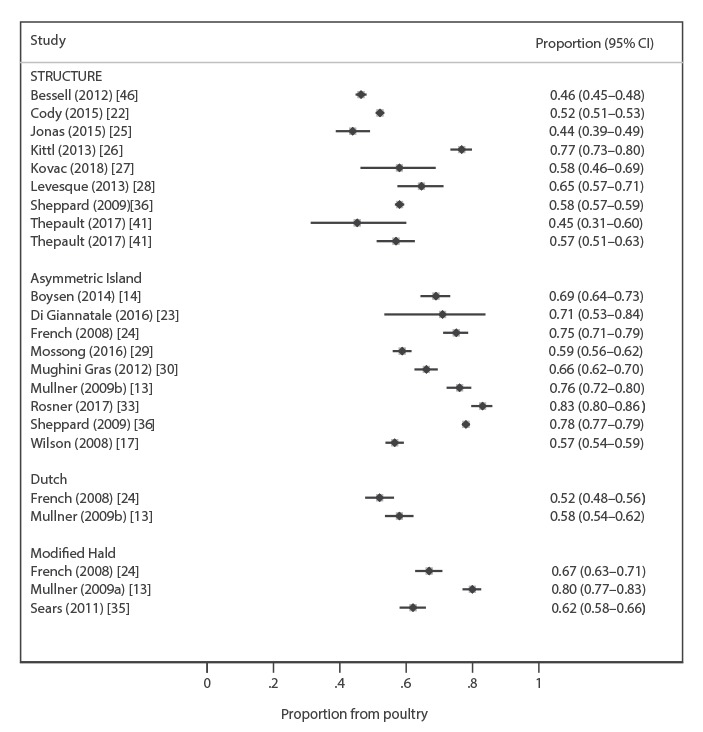
Forest plot of the proportion of *Campylobacter jejuni* clinical isolates attributed to poultry by different studies, and uncertainty around these estimates

Attribution of human *C. jejuni* isolates to poultry using seven-locus MLST by the AI model ranged between 57% and 83% [13,14,17,23,24,29,30,33,36]; by STRUCTURE between 44% and 77% [22,25-28,36,41,46]; the Dutch model between 52% and 58% [13,24]; the Hald model 52% [14]; and the MH model between 62% and 80% [13,16,24,35] (Figure 2 and Supplementary Table). In all four studies reporting results from *C. jejuni* datasets using more than one attribution algorithm, the AI model attributed higher proportions to poultry than other methods [13,14,24,36]. The one study using STRUCTURE analysis of 15 alternative *C. jejuni* loci attributed 57% to poultry [41]. The variation across estimates for attribution to poultry among studies, as determined by the I^2^ index, was greater than 90% for both AI and STRUCTURE analyses, showing substantial between-study variation so that a single summary estimate of results by method or overall is not supported (Figure 2).

CI: confidence interval.

I^2^ index > 90% for STRUCTURE and Asymmetric Island models so no summary estimates are given.

Between 38.0% and 82.4% of human *C. coli* were estimated to come from poultry in studies using the AI model [29,30,32,33,36,37], and between 40.0% and 86.4% using STRUCTURE [25,26,34,36].

Where both *C. jejuni* and *C. coli* species results were reported together, the AI model attributed between 68% and 77% to poultry [31,38] and STRUCTURE identified a range of between 19% and 48.7% [39,40]. Studies using the AI model were found to attribute significantly more *C. jejuni* isolates to poultry than STRUCTURE (p = 0.007), but no difference was detected for *C. coli* isolates attributed to poultry by these two methods.

In all articles that reported the attribution of *C. jejuni* to poultry, cattle and other sources, using seven-locus MLST, cattle were the second most predominant source identified by all methods with the exception of one STRUCTURE and one AI analysis that identified sheep [22] and pets [33] as the second most prevalent sources, respectively. Where cattle and sheep were considered together they were the second most prevalent *C. jejuni* source identified regardless of the algorithm used in three seven-locus MLST studies [21,29,36].

Ruminants were identified as the predominant source of clinical *C. coli* in one AI analysis [32], and sheep in one STRUCTURE analysis [34], while equal proportions of disease were attributed to poultry and sheep in a further STRUCTURE analysis [36]. In the five remaining AI [29,30,33,36,37] studies and one STRUCTURE [25] study considering the sources of human *C. coli* infection, poultry were the predominant source, followed by either cattle or a combined ruminant class.

The two studies that reported AI analyses of combined *C. jejuni* and *C. coli* datasets [31,38] both identified poultry as the primary and cattle as the secondary sources of disease. One STRUCTURE analysis of both species, comparing attribution at three time periods, reported poultry as the main disease source, with the secondary source changing from cattle to sheep in the final study period [40]. A further STRUCTURE analysis investigating the sources of *C. jejuni* and *C. coli* in children in urban and rural settings found poultry to be the predominant source followed by cattle in urban areas, but cattle as the prevalent and wild birds as the secondary sources in rural areas [39].

## Discussion

This review supports poultry and ruminants as the main sources of human campylobacteriosis across the settings investigated, with more than half of human campylobacteriosis cases attributed to poultry. Studies varied in the populations investigated, algorithms used, and approaches to choosing reference datasets for potential sources, but consistently identified the importance of poultry as a source. All studies were from high-income countries, with a substantial evidence gap for low- and middle-income countries.

Between-study comparisons were limited by the wide range of approaches used. Many enhanced the size of reference populations by access to publicly available datasets alongside more limited local data, sometimes using source data distant from the human isolate datasets, while others were limited to smaller reference datasets closer in time and place to the human infections. Although host-associated genetic signal has been shown to be stronger than the effects of geography [20], geographical distance among isolates from a single host source has the potential to cause bias in attribution analyses [20,47]. Smid and colleagues [38] investigated a range of factors affecting the outcome of AI attribution and identified that the inclusion of non-contemporaneous data and data from other countries reduced the attribution of Dutch disease isolates to chicken. Other authors used principal component analysis, to determine the most suitable countries of isolate origin for inclusion in attribution datasets [30,31,33]. Temporal separation between isolates may also create bias, with more pronounced effects reported for *C. coli* than *C. jejuni* populations [26]. There is no definitive evidence on the relative benefits of having larger more diverse reference datasets from potential sources of infection, or smaller ones closer to the human case isolates in time and place.

The number and composition of sources considered also varied across studies. Environmental isolates were considered as a proxy for other wildlife sources in some, but frequently included water samples that can be contaminated by farm slurry, agricultural run-off, or the disposal of abattoir effluent, as well as by wild animals [28,30]; indeed, ruminants have been implicated in cases of drinking water contamination by these means [48]. Datasets acquired from publicly available database collections rarely detailed sample type and often included samples from more than one point in the food chain [13,14,17,23,25,26,30,31,33-38,41,46,49,50]. Since all genotypes do not survive food processing procedures equally, broiler farm samples may, for example, represent the exposure of most individuals to *Campylobacter* from chicken less accurately than samples from retail poultry [51]. A combined ruminant (cattle and sheep) source population rather than separate cattle and sheep datasets was used in some studies but not in others, further limiting comparability among investigations. These ruminant species may host substantially overlapping *Campylobacter* populations [52].

The seven-locus MLST data used were primarily acquired using techniques described in the original publication of this methodology [8], but which have now been largely superseded as whole genome sequence (WGS) data are more common. Despite this change, the backwards compatibility of the gene-by-gene approach to WGS analysis permits extraction of the relevant internal gene fragment alleles for use in existing attribution methodologies, and also facilitates the identification of additional genes for use in such algorithms [53]. To date there has been only one publication detailing attribution using alternative loci identified from WGS, but as this study did not make any comparison with seven-locus MLST it was not possible to determine whether the novel methodology improved the accuracy of attributing generalist genotypes [41].

Comparison of results from different attribution models analysing the same datasets identified that the choice of model may be important. Results from the AI and STRUCTURE algorithms demonstrated that the AI model attributed more human *Campylobacter* infection to poultry, whereas the STRUCTURE algorithm attributed a higher proportion to ruminants [36]. These observations were confirmed by assessment of data from across all *C. jejuni* studies using these two algorithms where AI attributed a larger proportion to poultry.

The same pattern of results was observed when the AI, MH, and Dutch models were used to analyse highly similar *C. jejuni* datasets in two publications [13,43]; in all analyses poultry was predominant but the extent of this was more extreme with AI than with the MH or Dutch algorithms. In addition, the Dutch model identified environmental and wild-bird sources as causing a greater number of disease cases than either the AI or MH models.

Only in five studies was the accuracy of the attribution approach tested. Some evidence suggested that AI self-attributed poultry more accurately than STRUCTURE, although there is also grey literature reporting the opposite [54]. No study described adjustment of the raw results obtained in attributing human isolates for the biases estimated in self-attribution tests.

This systematic review brings together compelling evidence for poultry as the major source of human campylobacteriosis, with consistent results across several countries, time periods, and using different analytical algorithms and approaches to assembling isolate data from potential sources. The studies were mainly from Europe and New Zealand and highlight the gap in evidence for low and middle-income countries where *Campylobacter* may have a particularly large health burden [3]. This review also shows marked limitations as regards quality and comparability, with most studies not assessing their own accuracy. Moreover, none of the studies that measured accuracy and bias used this to adjust estimates of the proportion of human infection from each potential source or performing sensitivity analysis. The lack of evolution towards agreed optimal methods in the context of almost all studies using the same MLST data is striking. As WGS data become increasingly available, allowing the use of different genetic data across studies, moving to a consistent or optimum approach may be even more difficult although important to ensure comparability. The performance of tests of accuracy and bias such as self-attribution, and sensitivity analyses to take account of imperfect source attribution will be even more important. We recommend that validations, using approaches such as attribution of isolates from known sources, and adjustment for biases that are identified should be included in future population genetic source attribution studies and reports.

## Supplementary Data

400000 Supplementary Table
