# Supplementary material for: A systematic review of source attribution of human campylobacteriosis using multilocus sequence typing
Source: Euro Surveill. 2019 Oct 24;24(43):1800696. doi: 10.2807/1560-7917.ES.2019.24.43.1800696 (PMC6820127; doi:10.2807/1560-7917.ES.2019.24.43.1800696)
Supplement: Supplementary Table [file 1800696_MCCARTHY_SupplementaryTable.pdf]

This supplementary material is hosted by Eurosurveillance as supporting information alongside the article “A systematic review of source attribution of human campylobacteriosis using multi-locus sequence typing”, on behalf of the authors, who remain responsible for the accuracy and appropriateness of the content. The same standards for ethics, copyright, attributions and permissions as for the article apply. Supplements are not edited by Eurosurveillance and the journal is not responsible for the maintenance of any links or email addresses provided therein."

Supplementary Table. Summary of published studies using multi-locus sequence typing for source attribution

Attribution Model	Source Animal Dataset	Clinical Dataset	Species	Attribution to Poultry (%)	Attribution to Other Sources (%)	>1 sample type per source	Self-attribution analysis (%)	Reference (First author/year)
STRUCTURE	Chicken 1,288<br>Cattle 586<br>Sheep 249<br>Wild bird 170<br>Environmental 91<br>from UK, USA, Europe, New Zealand<br>1990 – 2006	3,451 from Scotland<br>2005-2006	*C. jejuni*	46.4	31.0 ruminant<br>1.9 wild bird	Yes	No	Bessell (2012)
Asymmetric Island	Chicken 185<br>Cattle 171<br>Duck 70<br>Turkey 96<br>Pig 4<br>from Denmark<br>Chicken 137<br>from other EU countries<br>2007 – 2008	406:<br>246 from Denmark<br>109 travel-related<br>51 unknown travel history<br>2007 - 2008	*C. jejuni*	52.0 Danish<br>17.0 imported	17.0 cattle<br>5.0 turkey<br>2.0 duck<br>7.0 pig	Yes	No	Boysen (2014)
Hald Model	Chicken 185<br>Cattle 171<br>Duck 70<br>Turkey 96<br>Pig 4<br>from Denmark<br>Chicken 137<br>from other EU countries<br>2007 – 2008	406:<br>246 from Denmark<br>109 travel-related<br>51 unknown travel history<br>2007 - 2008	*C. jejuni*	38.0 Danish<br>14.0 imported	16.0 cattle<br>6.0 turkey<br>3.0 duck<br>0.0 pig	Yes	No	Boysen (2014)
STRUCTURE	Chicken 742<br>Cattle 582<br>Sheep 217<br>Wild bird 921<br>from UK, Sweden, Australia<br>1999 – 2006	5,618 from UK<br>2003 - 2013	*C. jejuni*	47.1 - 56.7	15.0 - 20.5 cattle<br>25.0 - 31.1 sheep<br>2.1 - 3.5 wild bird	No	No	Cody (2015)

Asymmetric Island	583 from Italy<br>6,854 from PubMLST (European)<br>na	31 from Italy<br>2012	*C. jejuni*	69.8	8.3 cattle<br>5.3 small ruminant<br>7.3 wild bird<br>6.3 environmental<br>3.0 pork	Yes	No	Di Giannatale (2016)
Asymmetric Island	Chicken 275<br>Cattle 95<br>Sheep 136<br>Environmental water 92<br>Wild bird 22<br>from New Zealand<br>2005 - 2008	502 from New Zealand<br>2005 - 2008	*C. jejuni*	75.0	17.0 cattle<br>4.0 sheep<br>2.0 wild bird<br><1.0 water	Yes	No	French (2008)
Dutch Model	Chicken 275<br>Cattle 95<br>Sheep 136<br>Environmental water 92<br>Wild bird 22<br>from New Zealand<br>2005 – 2008	502 from New Zealand<br>2005 - 2008	*C. jejuni*	52.0	17.0 cattle<br>10.0 sheep<br>5.0 wild bird<br>11.0 water	Yes	No	French (2008)
Modified Hald	Chicken 275<br>Cattle 95<br>Sheep 136<br>Environmental water 92<br>Wild bird 22<br>from New Zealand<br>2005 – 2008	502 from New Zealand<br>2005 - 2008	*C. jejuni*	67.0	23.0 cattle<br>8.0 sheep<br>1.0 wild bird<br><1.0 water	Yes	No	French (2008)
STRUCTURE	Chicken 435<br>Cattle 73<br>Dog 134<br>from Switzerland and other countries<br>2003 – 2014	351 from Switzerland<br>2009	*C. jejuni*	44.0	36.0 cattle<br>20.0 dog	Yes	No	Jonas (2015)
STRUCTURE	Chicken 175<br>Cattle 24<br>Pig 256<br>from Switzerland and other countries<br>2008 – 2014	32 from Switzerland<br>2009	*C. coli*	76.0	16.0 cattle<br>8.0 pig	Yes	No	Jonas (2015)

STRUCTURE	Chicken 435<br>Dog 159<br>from Switzerland<br>2002 – 2012	649 from Switzerland<br>2002 - 2012	*C. jejuni*	76.8	23.2 dog	Yes	Yes;<br>62.5 chicken<br>38.1 dog	Kittl (2013)
STRUCTURE	Chicken 175<br>Pig 360<br>from Switzerland<br>2002 - 2012	81 from Switzerland<br>2002 - 2012	*C. coli*	86.4	13.6 pig	Yes	Yes;<br>79.8 chicken<br>70.1 pig	Kittl (2013)
STRUCTURE	Poultry 162<br>Cattle 40<br>Environmental 12<br>Slovenia, Austria, Germany<br>2002 – 2014	69 from Slovenia, Austria, Germany<br>2002 - 2014	*C. jejuni*	58.0	34.8 cattle<br>7.2 environmental	Yes	No	Kovac (2017)
STRUCTURE	Chicken 257<br>Cattle 87<br>Wild bird 63<br>Environmental water 266<br>from Canada<br>2005 – 2007	178 from Canada<br>2005 - 2007	*C. jejuni*	64.5	25.8 cattle<br>7.4 water<br>2.3 wild bird	No	No	Levesque (2013)
Asymmetric Island	Poultry 178<br>Cattle 98<br>Sheep 4<br>Goats 3<br>Pigs 2<br>Environmental water 207<br>from Luxembourg, The Netherlands, Belgium and France<br>2003 – 2014	1,153 from Luxembourg<br>2010 - 2013	*C. jejuni*	58.8	36.3 ruminant<br>4.9 environmental<br>0.2 swine	Yes	No	Mossong (2016)
Asymmetric Island	Poultry 150<br>Cattle 1<br>Goats 1<br>Pigs 60<br>Environmental water 122<br>from Luxembourg, The Netherlands, Belgium and France<br>2003 – 2014	136 from Luxembourg<br>2010 - 2013	*C. coli*	82.4	8.8 ruminant<br>4.5 environmental<br>4.4 swine	Yes	No	Mossong (2016)

Asymmetric Island	960 from The Netherlands, UK, Scotland and Switzerland - not detailed by species<br>1990 – 2006	696 from The Netherlands<br>2002 - 2003	*C. jejuni*	66.1	21.2 cattle<br>10.2 environmental<br>2.4 sheep<br>0.01 pig	Yes	No	Mughini Gras (2012)
Asymmetric Island	As above – not detailed by species	41 from The Netherlands<br>2002-2003	*C. coli*	70.0	12.2 cattle<br>8.9 environmental<br>5.0 sheep<br>4.9 pig	Yes	No	Mughini Gras (2012)
Asymmetric Island	Chicken 625<br>Cattle 168<br>Sheep 168<br>Pigs 160<br>Pets 133<br>Environmental 289<br>from The Netherlands, UK, Switzerland<br>1990 – 2006	737 from The Netherlands<br>2002 - 2003	*C. jejuni & C. coli*	77.0	18.0 cattle<br>2.0 environmental<br>2.0 sheep<br>1.0 pig	Yes	No	Mughini Gras (2013)
Modified Hald	Chicken 275<br>Cattle 95<br>Sheep 136<br>Environmental water 92<br>Wild bird 22<br>from New Zealand<br>2005 – 2008	773 from NZ<br>2005 - 2008	*C. jejuni*	80.0	10.0 cattle<br>9.0 sheep<br>4.0 environmental	Yes	No	Mullner (2009a)
Asymmetric Island	Chicken 275<br>Cattle 95<br>Sheep 136<br>Environmental water 92<br>Wild bird 22<br>from New Zealand<br>2005 – 2008	502 from New Zealand<br>2005 - 2008	*C. jejuni*	76.0	18.0 cattle	Yes	No	Mullner (2009b)
Modified Hald	Chicken 275<br>Cattle 95<br>Sheep 136<br>Environmental water 92<br>Wild bird 22<br>from New Zealand<br>2005 – 2008	502 from New Zealand<br>2005 - 2008	*C. jejuni*	>58.0 (58% from one poultry producer, total across 3 producers not given)	na	Yes	No	Mullner (2009b)

Dutch Model	Chicken 275<br>Cattle 95<br>Sheep 136<br>Environmental water 92<br>Wild bird 22<br>from New Zealand<br>2005 – 2008	502 from New Zealand<br>2005 - 2008	*C. jejuni*	58.0	19.0 cattle<br>12.0 environmental<br>11.0 sheep	Yes	No	Mullner (2009b)
Asymmetric Island	Poultry 108<br>Cattle 17<br>Sheep 32<br>Environmental water 11<br>from New Zealand<br>2009 - 2014	47 from<br>New Zealand<br>2005 - 2014	*C. coli*	38.0	55.0 ruminant<br>7.0 environmental	No	No	Nohra (2016)
Asymmetric Island	Chicken 1,081<br>Cattle 200<br>Other poultry 101<br>Pig 10<br>Pet 164<br>from Germany, Luxembourg, Switzerland and The Netherlands<br>2003 – 2014	536 from<br>Germany<br>2011 - 2014	*C. jejuni*	83.0	1.0 cattle<br>9.0 other poultry<br>16.0 pet<br>0.1 pig	Yes	No	Rosner (2017)
Asymmetric Island	Chicken 425<br>Pig 471<br>Pet 3<br>Cattle 7<br>Other poultry 59<br>from Belgium, France, Germany, Luxembourg, Switzerland, The Netherlands<br>2003 – 2011	76<br>2011-2014<br>460 from<br>Luxembourg<br>2003 - 2013	*C. coli*	56.0	0.4 cattle<br>32.0 pig<br>4.0 pet<br>8.0 other poultry	Yes	No	Rosner (2017)
STRUCTURE	Chicken 459<br>Cattle 85<br>Sheep 57<br>Pigs 322<br>from Scotland<br>2005 – 2006	307 from Scotland<br>2005 - 2006	*C. coli*	40.0	41.0 sheep<br>14.0 cattle<br>6.0 pig	Yes	No	Roux (2013)

Modified Hald	811:<br>Chicken<br>Cattle<br>Sheep<br>Environmental water<br>from New Zealand<br>2005 – 2008	572 from New Zealand<br>2005 - 2008	*C. jejuni*	62.0	25.6 cattle<br>8.8 sheep<br>3.2 water	Yes	No	Sears (2011)
Asymmetric Island	Chicken 1,288<br>Cattle 586<br>Sheep 249<br>Wild bird 170<br>Environmental 91<br>from UK, USA, Europe, New Zealand<br>1990 - 2006	4,743 from Scotland<br>2005 - 2006	*C. jejuni*	78.0	18.0 ruminant<br>4.0 wild bird & environment	Yes	Yes:<br>97 chicken<br>98 ruminant<br>77 wild bird<br>62 environmental	Sheppard (2009)
STRUCTURE	Chicken 1,288<br>Cattle 586<br>Sheep 249<br>Wild bird 170<br>Environmental 91<br>from UK, USA, Europe, New Zealand<br>1990 – 2006	4,743 from Scotland<br>2005 - 2006	*C. jejuni*	58.0	38.0 ruminant<br>4.0 wild bird & environment	Yes	Yes;<br>70 chicken<br>84 ruminant<br>54 wild bird<br>38 environmental	Sheppard (2009)
Asymmetric Island	Chicken 514<br>Cattle 98<br>Sheep 54<br>Pig 380<br>Turkey 110<br>Environmental 67<br>from UK, USA, Europe, New Zealand<br>1990 – 2006	504 from Scotland<br>2005 - 2006	*C. coli*	57.0	41.0 ruminant<br>1.0 pig<br>0.5 turkey<br>0.5 riparian	Yes	Yes;<br>na	Sheppard (2009)
STRUCTURE	Chicken 459<br>Cattle 86<br>Sheep 57<br>Pigs 322<br>Turkey 111<br>UK, USA, Europe, New Zealand<br>1990 – 2006	504 from Scotland<br>2005 - 2006	*C. coli*	40.0	40.0 sheep<br>14.0 cattle<br>6.0 pig<br><1.0 turkey	Yes	Yes;<br>na	Sheppard (2009)

Asymmetric Island	1,223 worldwide<br>2000 - 2008	500 from Scotland<br>2005 - 2006	*C. coli*	57.0	41.0 ruminant<br>1.0 swine<br>0.5 turkey<br>0.5 riparian	Yes	Yes;<br>95 chicken<br>89 ruminant<br>94 swine<br>92 turkey<br>88 riparian	Sheppard (2010)
Asymmetric Island	Chicken 400<br>Cattle 168<br>Sheep 160<br>Pig 133<br>Environmental 289<br>from The Netherlands, UK and Switzerland<br>1983 – 2007	1,208 from The Netherlands<br>2000 - 2011	*C. jejuni & C. coli*	68.0	24.0 cattle<br>6.0 environmental<br>2.0 sheep and pig	Yes	Yes;<br>89 chicken	Smid (2013)
STRUCTURE	680 from Scotland<br>2005 - 2006	202 from rural children Scotland<br>2000 - 2006	*C. jejuni & C. coli*	19.0	42.0 cattle<br>24.0 wild bird<br>12.0 sheep<br>3.0 pig	No	No	Strachan (2009)
STRUCTURE	680 from Scotland<br>2005 - 2006	76 from urban children Scotland<br>2000 - 2006	*C. jejuni & C. coli*	43.0	35.0 cattle<br>6.0 wild bird<br>15.0 sheep<br>1 pig	No	No	Strachan (2009)
STRUCTURE	84 from Scotland<br>2001	172 from Scotland<br>2001	*C. jejuni & C. coli*	47.7	19.5 cattle<br>11.2 sheep<br>19.6 wild bird<br>1.8 pig	No	No	Strachan (2013)
STRUCTURE	456 from Scotland<br>2005 – 2007	1,452 from Scotland<br>2005 - 2007	*C. jejuni & C. coli*	43.8	22.5 cattle<br>14.2 sheep<br>17.5 wild bird<br>0.03 pig	No	No	Strachan (2013)
STRUCTURE	708 from Scotland<br>2010 – 2012	1,292 from Scotland<br>2010 - 2012	*C. jejuni & C. coli*	48.7	12.7 cattle<br>26.8 sheep<br>9.2 wild bird<br>2.6 pig	No	No	Strachan (2013)

STRUCTURE*	Chicken 304<br>Cattle 43<br>Sheep 3<br>Wild bird 8<br>Environmental water 81<br>Duck 3<br>Dog 3<br>Goose 3<br>Other 52<br>from Europe and N. America<br>2001 – 2012	42 from France<br>2009	*C. jejuni*	45.8	46.9 ruminant<br>7.3 environmental	Yes	Yes;<br>80.7 chicken<br>68.2 ruminant	Thépault (2017)
STRUCTURE*	Chicken 304<br>Cattle 43<br>Sheep 3<br>Wild bird 8<br>Environmental water 81<br>Duck 3<br>Dog 3<br>Goose 3<br>Other 52<br>from Europe and N. America<br>2001 – 2012	281 from UK<br>2010 - 2011	*C. jejuni*	56.8	37.1 ruminant<br>6.1 environmental	Yes	Yes;<br>80.7 chicken<br>68.2 ruminant	Thépault (2017)
Asymmetric Island	Chicken 515<br>Cattle 282<br>Sheep 160<br>Pig 30<br>Wild bird 44<br>Rabbit 20<br>Sand 71<br>Water 23<br>from UK, Europe, Senegal,<br>USA, New Zealand<br>1990 – 2000	1,231 from NW<br>England<br>2000 - 2002	*C. jejuni*	56.5	35.0 cattle<br>4.3 sheep<br>0.8 pig<br>1.7 wild bird<br>0.6 rabbit<br>0.9 sand<br>0.2 water	Yes	No	Wilson (2008)

na: details not available.

* 15-locus MLST
